# Supplementary material for: A high-resolution mRNA expression time course of embryonic development in zebrafish
Source: eLife. 2017 Nov 16;6:e30860. doi: 10.7554/eLife.30860 (PMC5690287; doi:10.7554/eLife.30860)
Supplement: Supplementary file 6. [file elife-30860-supp6.zip › biolayout-clusters-files/Cluster239.html]

Cluster239


# Cluster239: Detail

### Go to ZFA detail

## GO

| GO ID | Description | Domain | Annotated | Expected | Observed | Adjusted p-value | Genes | Ensembl IDs |
| --- | --- | --- | --- | --- | --- | --- | --- | --- |
| GO:0005509 | calcium ion binding | molecular\_function | 389 | 0.22 | 4 | 0.0043 | notch1b celsr1b macf1b pcdh1g32 | ENSDARG00000052094 ENSDARG00000058259 ENSDARG00000089435 ENSDARG00000100829 |
